# 18-Month Effectiveness of Short-Course Antiretroviral Regimens Combined with Alternatives to Breastfeeding to Prevent HIV Mother-to-Child Transmission

**DOI:** 10.1371/journal.pone.0001645

**Published:** 2008-02-20

**Authors:** Valériane Leroy, Didier K. Ekouevi, Renaud Becquet, Ida Viho, Laurence Dequae-Merchadou, Besigin Tonwe-Gold, François Rouet, Charlotte Sakarovitch, Appolinaire Horo, Marguerite Timité-Konan, Christine Rouzioux, François Dabis, for the ANRS 1201/1202 DITRAME PLUS Study Group

**Affiliations:** 1 French National Institute for Health and Medical Research (INSERM), Unité 897, Centre de recherche "Epidémiologie et Biostatistique", Bordeaux, France; 2 Institut de Santé Publique, Epidémiologie et Développement (ISPED), Université Victor Segalen Bordeaux 2, Bordeaux, France; 3 Agence Nationale de Recherches sur le Sida (ANRS) 1201/1202 DITRAME PLUS Project, Programme PACCI, Centre Hospitalier Universitaire de Treichville, Abidjan, Côte d'Ivoire; 4 Centre de Diagnostic et de Recherches sur le SIDA (CeDReS), Centre Hospitalier Universitaire de Treichville, Abidjan, Côte d'Ivoire; 5 Service de Gynécologie-Obstétrique, Centre Hospitalier Universitaire de Yopougon, Abidjan, Côte d'Ivoire; 6 Service de Pédiatrie, Centre Hospitalier Universitaire de Yopougon, Abidjan, Côte d'Ivoire; 7 Laboratoire de Virologie Médicale, Centre Hospitalier Universitaire Necker Enfants Malades, Paris, France; Instituto de Pesquisa Clinica Evandro Chagas, FIOCRUZ, Brazil

## Abstract

**Objective:**

We assessed the 18-month effectiveness of short-course (sc) antiretroviral peripartum regimens combined with alternatives to prolonged breastfeeding to prevent mother-to-child transmission (MTCT) of HIV-1 in Abidjan, Côte d'Ivoire.

**Methodology:**

HIV-1 infected pregnant women received from ≥32–36 weeks of gestation scZidovudine (ZDV)+/−Lamivudine (3TC)+single-dose Nevirapine (sdNVP) at delivery within the ANRS 1201/1202 DITRAME-Plus cohort (2001–2003). Neonates received a sdNVP+7-day ZDV prophylaxis. Two infant-feeding interventions were systematically offered free of charge: formula-feeding or exclusive shortened breastfeeding with early cessation from four months. The reference group was the ANRS 049a DITRAME cohort (1994–2000) exposed to scZDV from 36 weeks, then to prolonged breastfeeding. Pediatric HIV infection was defined by a positive plasma HIV-1 RNA at any age, or if aged ≥18 months, a positive HIV-1 serology. Turnbull estimates of cumulative transmission risks (CTR) and effectiveness (HIV-free survival) were compared by exposure group using a Cox model.

**Findings:**

Among 926 live-born children enrolled, 107 (11.6%) were HIV-infected at 18 months. CTRs were 22.3% (95% confidence interval[CI]:16–30%) in the 238 ZDV long-term breastfed reference group, 15.9% (CI:10–27%) in the 169 ZDV+sdNVP shortened breastfed group; 9.4% (CI:6–14%) in the 195 ZDV+sdNVP formula-fed group; 6.8% (CI:4–11%) in the 198 ZDV+3TC+sdNVP shortened breastfed group, and 5.6% (CI:2–10%) in the 126 ZDV+3TC+sdNVP formula-fed group. Each combination had a significantly higher effectiveness than the ZDV long-term breastfed group except for ZDV+sdNVP shortened breastfed children, ranging from 51% (CI:20–70%) for ZDV+sdNVP formula fed children to 63% (CI:40–80%) for ZDV+3TC+NVPsd shortened breastfed children, after adjustment for maternal eligibility for antiretroviral therapy (ART), home delivery and low birth-weight. Substantial MTCT risk reductions are reachable in Africa, even in short-term breastfed children. The two sc antiretroviral combinations associated to any of the two infant feeding interventions, formula-feeding and shortened breastfeeding, reduce significantly MTCT with long-term benefit until age 18 months and without increasing mortality.

## Introduction

In African randomized clinical trials, several short-course (sc) or single-dose (sd) antiretroviral (ARV) regimens administered around the time of delivery (peripartum) have demonstrated a short-term efficacy to prevent mother-to-child transmission (PMTCT) of HIV-1, as estimated at age 6–8 weeks of life [1], [2], [3], [4]. An African pooled meta-analysis suggested the greater efficacy of ARV combinations than with any single ARV drug strategy [5]. All these scARV regimens using zidovudine (ZDV), lamivudine (3TC) or nevirapine (NVP) have been recommended for public health use in HIV-infected women and neonates in World Health Organization (WHO) evidence-based guidelines since 2000 [6].

However, in 2007, it is also well acknowledged that the subsequent risk of postnatal transmission of HIV-1 via breastfeeding is high in African populations where prolonged breastfeeding is the norm and may reduce the long-term efficacy of peripartum ARV regimens [6]. This long-term efficacy was shown to be still significantly sustained although lowered at age 24 months for scZDV [7] and at age 18-month for sdNVP but little benefit remained at 18-month using different regimens of scZDV+3TC [3]. It is therefore crucial to assess the long–term endpoints to fully measure ARV efficacy in breastfed populations. In addition, scARV regimens to prevent peripartum transmission should be complemented by postnatal interventions to reduce HIV transmission through breastfeeding and maximize their long-term combined efficacy. Among the different postnatal interventions, alternatives to prolonged breastfeeding, such as formula-feeding or exclusive breastfeeding with early cessation are two conceivable options in African urban settings where clean water is widely available. In these settings, field effectiveness of these interventions could be defined using a combined outcome, HIV-free survival, to assess their field efficacy in reducing MTCT taking into account the risk of alternatives to prolonged breastfeeding.

From March 2001 to March 2003, before the antiretroviral therapy access, we enrolled an open-label cohort study in Abidjan, Côte d'Ivoire to evaluate the acceptability, tolerance and field efficacy of a PMTCT package systematically offered to HIV-1 infected pregnant women: 1/two peripartum ARV interventions were successively introduced: ZDV+sdNVP during labor, followed by a short neonatal prophylaxis of ZDV+sdNVP; or the same regimen with the addition of a maternal 3TC [8]; 2/two post-partum infant feeding options were simultaneously offered: exclusive formula-feeding or shortened exclusive breastfeeding during four months [9], [10], [11]. In this paper, we report on the 18-month effectiveness (HIV-free survival) of these two combined PMTCT interventions; and compare them to an historical group followed in the same population and exposed to ZDV only with unrestricted prolonged breastfeeding.

## Methods

### Study sites

The ANRS 1201/1202 Ditrame-Plus project was conducted in community-run facilities in two urban districts of Abidjan, Côte d'Ivoire where HIV-1 seroprevalence in pregnant women was estimated to be around 11% in 2001–2003 [12].

### Study design

The study design was a non-randomized open-label prospective cohort [8], [9], [10], [11]. The peripartum ARV intervention was followed by one of the two infant feeding options then completed by the case management of HIV-infected children including cotrimoxazole prophylaxis. No maternal or pediatric antiretroviral therapy (ART) other than the study drugs for PMTCT indications was available at that time in Côte d'Ivoire. The study protocol was approved by the Ethics Committee of the National AIDS Control Program in Côte d'Ivoire and the Institutional Review Board of the French Agence Nationale de Recherches sur le SIDA (ANRS).

### Inclusion procedures

All consenting pregnant women attending the selected prenatal clinics, living within the city limits, presenting at <36 weeks of gestation, age ≥18 years, HIV-1 or HIV-1+2-infected, with hemoglobinemia ≥7 g/dl, and having signed an informed consent were systematically included [8], [13], [14].

### Study interventions

Women included received subsequently a peripartum ARV regimen as follows [8]: from 2001 to 2002, oral ZDV from 36 weeks of gestation daily and sdNVP at beginning of labor followed by a neonatal post-exposure prophylaxis: directly observed sdNVP on Day 2 and a seven-day course of ZDV syrup; from 2002 to 2003, the same maternal regimen was given from 32 weeks of gestation with the addition of 3TC. The ZDV+3TC maternal regimen was continued for three days post-partum. The neonatal regimen remained unchanged. Women received a supplementation in multivitamins [15], iron and folates, and malaria chemoprophylaxis according to Ivorian guidelines.

At inclusion, all pregnant women were individually counseled about both the advantages of breast-milk and the risk of HIV postnatal transmission. They were then systematically offered two alternative options to prolonged breastfeeding: exclusive formula-feeding from birth (with a drug inhibiting lactation) or exclusive shortened breastfeeding with early cessation within the fourth month, described elsewhere [9], [10], [11]. All women could express their infant feeding choice until birth and the staff supported their choice and counseled them accordingly. These two options were proposed free of charge including a controlled distribution of local powdered infant formula (one box at each follow-up visit) from birth or the date of weaning until nine months of age. Equipment was provided free of charge to women willing to refrain from breastfeeding: bottles, teats, cups, bottle brushes, pans to sterilize, thermos to keep safely clean water. Women were also trained to prepare and practice correctly formula-feeding [16]. Cup-feeding was encouraged after four months of age. In March 2003, because of the political turmoil in Côte d'Ivoire, we stopped offering the formula-feeding option, considering the risks of breakdown distribution of the breast milk substitutes were ethically unacceptable and we oriented every new woman to the shortened breastfeeding option until the end of the project, except if she deliberately decided to formula-fed her child. A systematic vitamin A supplementation was provided to children according to WHO recommendations. HIV-infected children received a cotrimoxazole prophylaxis systematically from six weeks of age until at least their first birthday [17].

### Follow-up procedures

Two clinics were exclusively dedicated to the follow-up of the 800 mother-infant pairs, with 60 health care workers recruited from the local area and specifically trained for this program. Pregnant women were followed weekly until delivery and mother-infant pairs were seen 48 hours after birth, weekly until six weeks of age, monthly until nine months of age, and every three months until their second birthday. At each scheduled visit, clinical, nutritional, psychosocial and biological follow-up of both mothers and infants was proposed. Four nutritionists individually counseled the women about safe infant feeding practices whenever needed. Care services were also available whenever needed between scheduled visits. All transport costs were reimbursed and all care expenses related to any scheduled visit or clinical event were entirely supported by the project.

Pediatric blood samples were taken for pediatric HIV-1 diagnosis at Day 2, week 4–6, then three monthly until one year, month 18 and 24 and two months after complete cessation of breastfeeding if any. A pediatric HIV-1 infection case was initially diagnosed using a commercial plasma HIV-1 RNA assay (Versant bDNA HIV RNA kit version 3.0, Bayer diagnostics, Emeryville, CA, USA) [18], then from 2003, a TaqMan HIV-1 RNA real-time PCR test was used for this purpose, with a threshold set up to preserve a 100% inter-assay reproducibility [19]. Pediatric HIV-1 infection was defined as a positive plasma HIV RNA at any age, or if aged ≥18 months, a positive HIV serology. Peri-partum transmission was defined in a child with a negative PCR at Day 2 who was later positive at 4–6 weeks. Postnatal transmission (PT) was defined as a child with a negative HIV-1 PCR from a sample obtained at age >30 days who later became infected. At the end of follow-up, children were no longer at risk for HIV infection when they were either HIV-1 infected or uninfected while complete cessation of breastfeeding was obtained for at least two months.

### Data collection

At each scheduled visit, clinical events that occurred in both mothers and children since the last visit were documented and infant feeding practices were recorded via structured questionnaires by trained social workers who were not involved in nutritional counseling [9]. Infants were classified at each scheduled visit as breastfed, mixed-fed or formula-fed using 7-day recall histories [20]. We used the WHO definitions to allow a better comparability of results between studies [21], [22].

### Comparison group

We used for reference the pooled data of two consecutive series of mother-infant pairs, first those randomized to the ZDV arm of the ANRS 049a trial from 1995 to 1998 in Abidjan [1] and second those receiving ZDV in an open-fashion on the same sites in 1999–2000 [23]. Maternal ZDV was initiated at 36 weeks and continued for seven days post-delivery. No ZDV was given to neonates. No infant feeding intervention was promoted and prolonged and unrestricted breastfeeding was the norm [7], [24].

### Statistical analysis

Every HIV-1 infected pregnant woman enrolled was eligible if she gave a live-birth. The allocation to an ARV intervention was on an intent-to-treat basis. The allocation to one infant feeding group (formula-fed or shortened breastfed) was based on the actual feeding option implemented at Day 2 after delivery whatever the prenatal choice, as some of the mothers did not express their infant feeding choice before delivery and some of the children were not fed in their first 24 hours of life.

Cumulative transmission risks (CTRs) of paediatric HIV infection or (HIV or death) combined were estimated using Turnbull method for each peripartum scARV regimen and in each infant feeding group defined at ages 6-week, 6-month and 18-month in an intent-to-feed analysis [25]. The analysis was also stratified according to maternal eligibility criteria for ART using 2003 WHO criteria (WHO clinical stage 4 or WHO clinical stage 3 and CD4<350/mm^3^ or WHO clinical stage 1–2 and CD4<200/mm^3^) [26]. Results were expressed in percentages with their 95% Confidence Interval (CI). When Turnbull CIs were not computable, Kaplan-Meier estimates were used for CIs, which was satisfactory since intervals between tests were less than three-month [25].

For the HIV infection analysis, all first live-born fed at least once were included and death was a censoring event. For the HIV-free survival analysis, all first live-births were included and the time of the event was the earlier of infection or death. Infants who were lost-to-follow-up before reaching study end points were censored at their last HIV-negative test. The competing risk analysis usually recommended in breastfed children [25] was not applicable as part of the children was not breastfed at all.

Effectiveness was expressed as 1 minus the ratio of estimated CTR of HIV or death in the intervention group compared to the reference group. Determinants for 18-month infection or death were explored using a Cox model with the following variables: peripartum ARV regimen combined with infant feeding option, place and mode of delivery, maternal eligibility for ART, and low-birth weight as a proxi of prematurity. A stepwise backward multivariate analysis included all variables with p<0.25 in the univariate analysis. Our study protocol implied that the treatment was initiated earlier in the ZDV+3TC+sdNVP cohort (32 weeks) than in the ZDV+sdNVP cohort (36 weeks). As a consequence, the duration of prenatal prophylaxis was strongly correlated with the antiretroviral drug regimen. The variable ‘cohort’ used in the Cox model analysis took into account both the duration of prenatal prophylaxis and the antiretroviral drug regimen used. For this Cox model, time to infection was estimated to the first positive test or the mid-point between the last negative test and the first positive test when possible. All statistical analyses were carried out with the SAS software (version 9.1; SAS Institute, Inc, Cary, NC, USA).

## Results

### Study population

Between March 6th 2001 and July 31st 2003, 808 women were included in the ANRS 1201/1202 Ditrame-Plus cohorts (Table 1). After exclusion of pregnant women lost-to-follow-up prior to delivery, multiple birth outcomes, and stillbirths, 711 first live-born children were enrolled: 375 received ZDV+sdNVP and 336 ZDV+3TC+sdNVP and were either formula-fed or short-term breastfed. From September 1995 to 1998, 249 first live-born children whose mother received ZDV alone were included in the historical cohort in the same Abidjan health facilities. Among those, only eight (3%) were formula-fed at that time and were excluded, leaving 241 children exposed to ZDV and long-term breastfeeding as the reference group. Overall, among the 952 first-live born enrolled, 24 with no blood sample available (2.5%) and two that could not be classified for infant feeding modalities at Day 2 were excluded from the present analysis (Table 1).

**Table 1:** Number of women and children by peri-partum short-course (sc) antiretroviral regimen and infant-feeding modality for prevention of Mother-To-Child transmission (PMTCT) of HIV.

Cohort (Period of inclusion)	DITRAME ZDV (1994–1999)	Ditrame-Plus ZDV+sdNVP (2001–2002)		Ditrame-Plus ZDV+3TC+sdNVP (2002–2003)		Total
Women enrolled	261	420		388		1069
Excluded (HIV-2 infection only or HIV not confirmed)	0	18		16		34
Lost-to-follow-up before delivery (%)	8 (3.0)	21 (5.0)		23 (5.9)		52
Women who gave birth	253	381		349		983
Multiple birth outcomes excluded (stillbirths)	3 (0)	21 (1)		17 (0)		41
Stillbirths (%)	4 (1.5)	6 (1.6)		13 (3.7)		23
Live births included in the analysis	241* (100%)	375 (100%)		336 (100%)**		952**
Mortality: number of deaths among live-born children (%)	33 (13.7)	35 (9.3)		22 (6.5)		90
Before Day 8	3	6		7		16
Day 8–Day 27	0	0		2		2
Day 28–Day 365	24	23		13		60
12 months–18 months	6	6		0		12
Not tested for paediatric HIV-1 infection (%)	3 (1.2)	11 (2.9)		10 (2.9)		24
Included in the HIV-transmission analysis	238 (100%)	364 (100%)		326 (100%) **		928**
HIV-1 infection (%)	47 (19.8) [100%]	40 (11.0) [100%]		20 (6.1) [100%]		107
Peri-partum infection (<week 4) [%]	28 [60]	25 [63]		17 [85]		70
Postnatal infection (≥week 4) [%]	15 [32]	13 [32]		2 [10]		30
Timing of infection unknown [%]	4 [8]	2 [5]		1 [5]		7
Infant feeding modality unknown	0	0		2		2
Included in the HIV-transmission analysis according to infant feeding modality	Long-term breastfed N = 238	Formula-fed N = 195	Shortened breastfed N = 169	Formula-fed N = 126	Shortened breastfed N = 198	926**
HIV-1 infected (%)	47 (19.8) [100%]	18 (9.2) [100%]	22 (13.0) [100%]	7 (5.6) [100%]	13 (6.6) [100%]	107
Peri-partum infection (<week 4) [%]	28 [60]	16 [89]	9 [41]	7 [100]	10 [77]	70
Postnatal infection (≥week 4) [%]	15 [32]	2 [11]	11 [50]	0 [0]	2 [15]	30
Timing of infection unknown [%]	4 [8]	0 [0]	2 [9]	0 [0]	1 [8]	7

ANRS 1201/1202 Ditrame-Plus and ANRS 049a DITRAME cohorts, Abidjan, Côte d'Ivoire.

ZDV = short-course zidovudine; sdNVP = single-dose nevirapine during labour; 3TC = lamivudine.

* Eight formula-fed children were excluded.

** Two children were not classified for infant feeding modality.

At baseline, women from the Ditrame-Plus cohorts were more advanced in HIV-disease progression compared to the ZDV historical cohort (Table 2): 17% of women in the Ditrame-Plus cohorts had CD4<200 while this proportion was 11% in the ZDV cohort. Similarly, the proportion of women eligible for ART and the plasma HIV-RNA viral load was significantly higher in the Ditrame-Plus cohorts than in the ZDV cohort. The median prenatal duration of ARV prophylaxis increased significantly over time, from 20 days in the ZDV cohort to 29 in the ZDV+sdNVP cohort and up to 50 in the ZDV+3TC+sdNVP cohort (Table 2). The proportion of women taking the ARV labour dose increased from 83% with ZDV alone to 93% with the two ARV combinations (p<10^−4^). There was no statistical difference between the three groups for place of delivery, the frequency of C-section and the frequency of low birth-weight. However, within the Ditrame-Plus cohorts, women who initiated breastfeeding delivered more frequently at home than those from the formula-fed group, 14.2% versus 5.6%, respectively in each ARV intervention cohort.

**Table 2:** Baseline and follow-up characteristics of pregnant women and children included in the long-term transmission risk analysis according to infant feeding modality.

	DITRAME ZDV N = 241	Ditrame-Plus ZDV+sdNVP N = 375			Ditrame-Plus ZDV+3TC+sdNVP N = 334†			p1	p2
Women who delivered a live-birth with infant feeding modality defined	Long-term breastfed N = 238	Formula-fed N = 195	Shortened breastfed N = 169	p	Formula-fed N = 126	Shortened breastfed N = 198	p		
Median age in years (IQR)	26 (23–30)	27 (24–31)	25 (22–30)	0.017	27 (24–30)	26 (23–31)	0.85	0.54	0.80
Median parity (IQR)	2 (1–3)	1 (1–3)	2 (1–3)	0.95	1 (0–2)	1 (1–2)	0.10	0.009	0.022
Primigravida (%)	23 (9.7)	12 (6.2)	19 (11.2)	0.08	7 (5.6)	14 (7.1)	0.59	0.36	0.31
Median gestational age (IQR) at enrolment	36 (36–36)	36 (36–37)	36 (36–37)	0.13	32 (32–34)	33 (33–34)	0.26	<0.0001	<0.0001
Median lymphocyte CD4 count/mm^3^ (IQR)	487 (307–705)	377 (229–563)	358 (260–493)	0.92	398 (252–602)	419 (265–564)	0.82	<0.0001	0.03
<200 CD4 cells/mm^3^ (%)	27 (11.3)	40 (20.5)	27 (16.0)	0.28	18 (14.3)	30 (15.1)	0.87	0.06	0.22
Indication for ART (2003 WHO criteria) *	36 (15.5)	62 (31.8)	41 (24.6)	0.13	26 (20.6)	40 (20.3)	0.94	<0.0006	0.015
Mean log10 HIV-1 RNA plasma viral load at enrolment (SD)	(n = 92) ** 4.05 (0.86)	4.14 (0.93)	4.01 (0.84)	0.18	4.35 (0.92)	4.43 (0.94)	0.52	<0.0001	<0.0001
Median duration of prepartum treatment in days (IQR)	20 (13–30)	30 (21–40)	28 (17–40)	0.21	51 (37–64)	50 (32–64)	0.42	<0.0001	<0.0001
Delivered at home (%)	36 (15.2)	11 (5.6)	24 (14.2)	0.006	7 (5.6)	28 (14.1)	0.015	0.10	0.61
C-section (%)	9 (3.8)	11 (5.6)	7 (4.1)	0.51	10 (7.9)	7 (3.5)	0.08	0.71	0.86
Low birth-weight (<2500 g) (%)	29 (12.3)	25 (12.8)	13 (7.7)	0.11	21 (16.7)	24 (12.1)	0.25	0.38	0.17

ANRS 1201/1202 Ditrame-Plus and ANRS 049a DITRAME cohorts, Abidjan, Côte d'Ivoire.

p, test for difference between formula-fed and shortened breastfed children within Ditrame-Plus cohorts of similar scARV regimen.

p1, test for difference between the three groups ZDV, ZDV+sdNVP, ZDV+3TC+sdNVP.

p2, test for difference between the ZDV+sdNVP and ZDV+3TC+sdNVP groups

IQR interquartile range; NA not applicable. 3TC = lamivudine; sdNVP = single-dose nevirapine during labour; ZDV = zidovudine. SD: standard deviation.

† Excluding two children not classified for infant feeding modality. *ART = antiretroviral therapy if WHO clinical stage 4 or WHO clinical stage 3 and CD4<350/mm^3^ or WHO clinical stage 1/2 and CD4<200/mm^3^.

** 4.8 log10 for a sub-sample of 20 transmitting mothers and 3.7 for a sample of 72 non-transmitting mothers.

The 238 ZDV children from the historical ZDV cohort were predominantly breastfed for a median duration of 7.3 months in the (IQR: 6–10) and defined as “long-term breastfed”. In this cohort predominant breastfeeding was the norm with early introduction of water from day one in median [9]. In Ditrame-Plus, among the 688 children included in this analysis, 321 (47%) children were formula fed of whom 47 (15%) women failed to maintain exclusive formula-feeding and breastfed at least once. In Ditrame-Plus, 367 (53%) children were short-term breastfed for a median duration of 3.9 months (IQR: 3–8) in scZDV+sdNVP children and 4.4 months (IQR: 4–7) in sc(ZDV+3TC)+sdNVP children. Infant feeding practices in Ditrame-Plus were extensively described elsewhere [9], [10]. When the formula feeding option was not offered any more to new women included from March 2003 to July 2003, we did not observe any modification in the patterns of breastfeeding in breastfed children (similar mixed feeding practices and exclusive breastfeeding behaviour). In the two Ditrame-Plus formula-feeding cohorts, 47 (15%) women failed to maintain exclusive formula-feeding and breastfed at least once. At the end of the follow-up, 96.2% of the formula-fed children were no longer at risk for HIV-1 infection while this proportion was only 84.7% in the three breastfed cohorts, because of the longer duration of breastfeeding (p<10^−6^).

### 18-month cumulative rates of HIV transmission

At age 18-month, 107 children were HIV-1 infected of whom 30 (28%) were postnatal transmission cases (two in the formula-fed groups), 70 (68%) were peripartum infections and seven (7%) remained of unknown timing. Crude CTRs of infection at 18-month using Turnbull estimates were 22.3% (95%CI: 16.1%–29.8%) in the 238 ZDV long-term breastfed reference group, 13.4% (95%CI: 8.9%–19.9%) in the ZDV+sdNVP cohort (irrespective of postnatal intervention) and 6.3% (95%CI: 3.8%–9.1%) in the ZDV+3TC+sdNVP cohort, also irrespective of postnatal intervention (Table 3). CTRs of infection at 18-month were lower in formula-fed children than shortened breastfed children, but this difference was less marked after ZDV+3TC+sdNVP peripartum exposure than after ZDV+sdNVP peripartum exposure. When stratifying the sample according to the 2003 WHO criteria for maternal ART eligibility, all CTRs of infection at 18-month were consistently 2 to 3 times higher among ART-eligible mothers than those who were not (Table 3).

**Table 3:** Estimated cumulative transmission risk (CTR) and 95% confidence interval (CI) of HIV-1 infection diagnosis at age 18 months in live-born children.

Cohort	DITRAME ZDV Long-term breastfed I/n = 47/238	Ditrame-Plus ZDV+sdNVP I/n = 40/364		Ditrame-Plus ZDV+3TC+sdNVP I/n = 20/326 **	
6 weeks	13.2 (7.2–17.4)	8.9 (5.3–12.3)		5.9 (3.4–8.5)	
6 months	18.9 (12.9–24.3)	9.7 (6.5–12.7)		6.3 (3.8–9.1)	
18 months	22.3 (16.1–29.8)	13.4 (8.9–19.9)		6.3 (3.8–9.1)	
Infant-feeding modality	Long-term breastfed	Formula-fed	Shortened breastfed	Formula-fed	Shortened breastfed
	I/n = 47/238	I/n = 18/195	I/n = 22/169	I/n = 7/126	I/n = 13/198
6 weeks	13.2 (7.2–17.4)	9.4 (4.8–13.6)	8.6 (2.9–13.2)	5.6 (1.6–9.6)†	6.2 (3.1–9.9)
6 months	18.9 (12.9–24.3)	9.4 (5.1–13.6)	10.1 (5.5–14.9)	5.6 (1.6–9.6)†	6.8 (3.6–10.7)
18 months	22.3 (16.1–29.8)	9.4 (5.5–13.6)	15.9 (9.6–26.5)	5.6 (1.6–9.6)†	6.8 (3.6–10.7)
Mothers eligible for ART*	I/n = 16/36	I/n = 10/62	I/n = 9/41	I/n = 3/26	I/n = 5/40
6 weeks	26.2 (11.4–40.9)†	16.3 (7.1–25.6)†	10.1 (0.7–19.6)†	11.5 (0–23.8)†	13.0 (2.3–23.7)†
6 months	41.6 (24.8–58.3)†	16.3 (7.1–25.6)†	15.9 (4.2–27.7)†	11.5 (0–23.8)†	13.0 (2.3–23.7)†
18 months	48.1 (30.9–65.2)†	16.3 (7.1–25.6)†	25.2 (10.8–39.6)†	11.5 (0–23.8)†	13.0 (2.3–23.7)†
Mothers not eligible for ART*	I/n = 29/196	I/n = 8/133	I/n = 13/126	I/n = 4/100	I/n = 8/157
6 weeks	9.3 (5.2–13.4)†	4.5 (1.0–8.0)†	4.9 (1.1–8.7)†	4.0 (0–7.9)†	4.0 (0.9–7.0)†
6 months	13.6 (8.7–18.5)†	6.2 (2.0–10.3)†	8.3 (3.4–13.2)†	4.0 (0–7.9)†	5.4 (1.8–9.1)†
18 months	15.5 (10.3–20.7)†	6.2 (2.0–10.3)†	11.1 (5.4–16.8)†	4.0 (0–7.9)†	5.4 (1.8–9.1)†

ANRS 1201/1202 Ditrame-Plus and ANRS 049a DITRAME cohorts, Abidjan, Côte d'Ivoire, 2001–2005. Turnbull estimates and Kaplan-Meier estimates.

I/n: number of children infected/number of children at risk for mother-to-child transmission.

* ART = antiretroviral therapy if WHO clinical stage 4 or WHO clinical stage 3 and CD4<350/mm^3^ or WHO clinical stage 1/2 and CD4<200/mm^3^.

** Two children were not classified for infant feeding modality. † Confidence interval based on Kaplan-Meier estimates.

**Table 4:** Determinants of 18-month HIV-1 infection or death (Cox proportional hazard model; reference: ZDV long-term breastfed).

Variables				Crude (Univariate)			Adjusted (Multivariate*)		
	N = 950	CTR^ §^	95% CI	HR#	95% CI	p^§§^	HRa	95% CI	p
Cohort #						0.003			0.0001
ZDV long-term breastfed (reference)	241	24.5	19–30	1	-		1	-	
ZDV+sdNVP shortened breastfed	177	18.4	12–24	0.72	0.5–1.3		0.68	0.4–1.1	
ZDV+sdNVP formula-fed	198	15.8	9–22	0.56	0.4–0.9		0.49	0.3–0.8	
ZDV+3TC+sdNVP shortened breastfed	203	10.4	6–15	0.41	0.2–0.7		0.37	0.2–0.6	
ZDV+3TC+sdNVP formula-fed	131	12.3	7–18	0.49	0.3–0.9		0.43	0.2–0.8	
Maternal CD4 count						<0.0001			
[0–200]	146	27.9	21–35	3.08	1.9–4.9				
[200–350]	234	20.4	15–26	2.08	1.3–3.3				
[350–500]	232	14.1	10–19	1. 39	0.9–2.3				
≥500	338	11.2	7–15	1	-				
Maternal clinical HIV stage						0.0034			
1–2	738	14.9	12–18	1	-				
3	207	23.0	17–29	1.69	1.2–2.4				
4	5	40.0	0–83	3.80	0.9–15.4				
Not eligible for ART	729	13.7	11–17	1	-		1	-	
Eligible for ART	212	27.0	21–33	2.25	1.6–3.1	<0.0001	2.54	1.8–3.6	<0.0001
Maternal plasma viral load (for one log increase)	-	-	-	2.47	2.0–3.1	<0.0001			
Maternal age (for +10 years)	-	-	-	1.14	0.9–1.5	0.39			
Prepartum prophylaxis						0.018			
<30 days	426	20.5	16–25	1	-				
30 days and more	524	13.8	11–17	0.68	0.5–0.9				
Parity (for one unit increase)	-	-	-	1.02	0.9–1.1	0.73			
Primigravida						0.77			
No	136	16.6	14–19	1	-				
Yes	13	19.1	10–28	1.09	0.6–1.9				
Gestational age at inclusion (for one week increase)	-	-	-	1.06	0.9–1.1	0.16			
Delivered at home						<0.0001			0.0005
No	839	14.9	12–18	1	-		1	-	
Yes	109	32.4	23–42	2.30	1.5–3.4		2.04	1.4–3.1	
C-section						0.80			
No	901	16.9	14–20	1	-				
Yes	47	15.2	5–26	0.91	0.4–1.9				
Low birth-weight (<2500 g)						0.0001			0.0002
No	825	15.1	12–18	1	-		1	-	
Yes	120	27.3	19–35	2.15	1.5–3.2		2.18	1.5–3.3	

ANRS 1201/1202 Ditrame-Plus and ANRS 049a DITRAME cohorts, Abidjan, Côte d'Ivoire.

*Variables with p<0.25 in the comparison of the three treatment groups (table 2). ^§^Estimated cumulative transmission risk (CTR) of HIV-1 infection at 18-month using Kaplan-Meier estimates. ^§§^Log-rank test; CI: confidence interval; ^#^univariate = unadjusted Cox proportional hazards model; HR: hazard ratio; HRa: adjusted Hazard Ratio; 3TC = lamivudine; sdNVP = single-dose nevirapine during labour; ZDV  = zidovudine. ART = highly active antiretroviral therapy if WHO clinical stage 4 or WHO clinical stage 3 and CD4<350 /mm^3^ or WHO clinical stage 1/2 and CD4<200/mm^3^. #in the adjusted analysis, this variable took into account both the treatment duration and the antiretroviral regimen used.

**Table 5:** Determinants of 18-month HIV-1 infection or death (Multivariate Cox proportional hazard model, reference: ZDV+sdNVP).

Variables				Crude (Univariate)			Adjusted (Multivariate*)		
	N = 709	CTR^ §^	95% CI	HR#	95% CI	p^§§^	HRa	95% CI	p
Cohort #						0.09			0.06
ZDV+sdNVP (reference)	375	16.8	13–21	1	-		1		
ZDV+3TC+sdNVP	334	11.1	8–15	0.70	0.5–1.1		0.66	0.4–1.0	
Infant feeding						0.87			0.46
Shortened breastfed (reference)	380	14.2	11–18	1	-		1		
Formula-fed	329	14.3	10–19	0.97	0.6–1.5		0.85	0.6–1.3	
Maternal CD4 count						0.0003			
[0–200]	119	23.6	16–31	3.75	2.0–7.1				
[200–350]	188	16.9	12–22	2.49	1.3–4.6				
[350–500]	180	11.8	7–17	1.67	0.9–3.3				
≥500	222	8.6	4–14	1	-				
Maternal clinical HIV stage						0.0007			
1–2	530	11.8	9–15	1	-				
3	174	20.7	15–27	1.98	1.3–3.0				
4	5	40.0	0–83	4.78	1.2–19.6				
Eligible for HAART	176	22.3	16–29	2.23	1.5–3.4	0.0001	1		0.0003
Not eligible for HAART	530	11.5	8–15	1	-		2.15	1.4–3.3	
Maternal viral load (for one log increase)	-	-	-	2.54	2.0–3.3	<.0001			
Maternal age (for +10 years)	-	-	-	1.11	0.8–1.6	0.59			
Prepartum treatment (for 10 days increase)				0.88	0.8–0.98	0.019			
Parity (1 increase)						0.56			
Primigravida						0.75			
No	653	14.0	11–17	1	-				
Yes	56	16.9	6–28	1.13	0.5–2.3				
Gestational age at inclusion (for one week increase)				1.02	0.9–1.1	0.73			
Delivered at home						0.0025			0.02
No	636	12.7	10–16	1	-		1		
Yes	73	27.4	17–38	2.22	1.3–3.7		1.88	1.1–3.2	
C-section						0.66			
No	671	14.0	11–17	1	-				
Yes	38	16.0	4–28	1.21	0.5–2.8				
Low birth-weight (<2500 g)						0.0002			0.0002
No	617	12.3	9–15	1	-		1		
Yes	91	25.6	27–35	2.48	1.5–4.0		2.50	1.5–4.0	

ANRS 1201/1202 Ditrame-Plus, 2001–2005, Abidjan, Côte d'Ivoire.

*Variables with p<0.25 in the comparison of the three treatment groups (table 2). ^§^Estimated cumulative transmission risk (CTR) of HIV or death at 18 months using Kaplan-Meier estimates (%). ^§§^logrank test; ^#^univariate analysis = unadjusted Cox proportional hazards model; CI confidence interval; HR hazard ratio; HRa adjusted Hazard Ratio; 3TC = lamivudine; NVPsd = single-dose nevirapine during labour; ZDV  = zidovudine. HAART = highly active antiretroviral therapy if WHO clinical stage 4 or WHO clinical stage 3 and CD4<350/mm^3^ or WHO clinical stage 1/2 and CD4<200/mm^3^. #in the adjusted analysis, this variable took into account both the treatment duration and the antiretroviral regimen used.

### 18-month cumulative rates of HIV transmission or death: effectiveness

Among the 24 children with an indeterminate HIV status, 16 died. Among the 950 children exposed to HIV or death and classified for infant modality, 149 were HIV-1 infected or dead at age 18-month. At 18-month, crude CTRs of infection or death using Turnbull estimates were 24.5% (95%CI: 19%–30%) in the 241 ZDV reference group, 16.8% (95%CI: 13%–21%) in the 375 ZDV+sdNVP group and 11.1% (95%CI: 8%–15%) in the 334 ZDV+3TC+sdNVP group.

### Determinants of 18-month effectiveness compared to the ZDV long-term breastfed cohort

The crude analysis of 18-month HIV transmission or death showed that the type of scARV regimen, low maternal CD4 count, advanced maternal clinical staging, maternal eligibility for ART, high maternal plasma viral load at inclusion, longer duration of pre-partum scARV prophylaxis, higher gestational age, home delivery, and low-birth weight, were significantly associated with HIV-infection or death (Table 4). The pre-partum scARV duration was highly correlated to the three ARV regimens, without any significant interaction (p ranging from 0.08 to 0.75). There was neither any interaction between ARV prophylaxis and gestational age (p ranging from 0.26 to 0.94), nor between treatment and home delivery (p ranging from 0.61 to 0.99). Using the ZDV long-term breastfed cohort as the reference (24.5% HIV transmission or death at 18 months), the protective effect of each of the scARV combinations was strong for all combinations except for the ZDV+sdNVP shortened breastfed children, with an adjusted non-significant effectiveness of 32% (0%–60%) just below the significant level. The adjusted effectiveness was significantly higher for the others combined peri-partum and post-partum regimens: 51% (20%–70%) for ZDV+sdNVP formula-fed children, 63% (40%–80%) for ZDV+3TC+sdNVP shortened breastfed children and 57% (20%–80%) for ZDV+3TC+sdNVP formula-fed children after adjustment for maternal eligibility criteria for ART, home delivery and low birth-weight in the final adjusted model (Table 4).

### Determinants of 18-month occurrence of HIV-infection or death within Ditrame-Plus

Within the Ditrame-Plus cohorts, the final model analyzing determinants of HIV-infection or death showed that the 3TC-containing regimen tended to be protective but was no statistically significant. In comparison with the ZDV+sdNVP as the reference, the adjusted protective effect of ZDV+3TC+sdNVP on long-term infection or death was not significant, 34% (95% CI:0%–60%, p = 0.06). Others risk factors of HIV-infection or death were the maternal ART eligibility criteria (aHR: 2.15; CI:1.4–3.3, p = 0.0003), home delivery (aHR: 1.88; CI:1.1–3.2, p = 0.02) and low-birth-weight (aHR: 2.50; CI:1.5–4.0, p = 0.0002) (Table 5). Formula-feeding was not a significant determinant of HIV-transmission or death compared to shortened breastfeeding (aHR: 0.85; CI:0.6–1.3, p = 0.46).

## Discussion

The absolute long-term efficacy of all scARV regimens administrated in peripartum in African breastfeeding populations is likely to be reduced beyond 4–6 weeks because of the breastfeeding HIV transmission [7]. We have previously reported on the short-term efficacy at age 6-week of two scARV combination regimens, ZDV+sdNVP and ZDV+3TC+sdNNVP [8]. Alternatives to prolonged breastfeeding are aimed to optimize the long-term efficacy of these peri-partum ARV regimens. We report here that a significant reduction of MTCT of HIV can be achieved, with these scARV interventions combined with two different alternatives to prolonged breastfeeding, with a long-term benefit sustained until age 18-month and without any increasing risk of death.

Several arguments justified the Ditrame-Plus study design. First, we chose a non-randomized approach to estimate the treatment effect as we considered there was *a priori* lack of equipoise between ZDV+sdNVP and ZDV alone. We hypothesized the addition of sdNVP to ZDV would be efficient by its partially different mechanism of action, and did not anticipate tolerance issues based on the HIVNET 012 trial findings [4]. In addition, as Ditrame-Plus women were enrolled and followed on the same sites than in the previous Ditrame trial cohort, we estimated that we would be able to control for the most important confounding factors and determinants of MTCT or death that were prospectively measured, such as maternal clinical and immunological HIV disease staging, mode of delivery, breastfeeding exposure and duration of the ARV prophylaxis. Second, we assessed the field effectiveness of the two infant feeding options in a sample representative of childbearing age women living in an urban African setting with water access. We chose a cohort design instead of a randomized clinical trial, to avoid the allocation of the infant feeding practices at random and then minimize the ethical considerations and the risk of non compliance inducing at-risk mixed feeding-practices, as previously reported [27], [28]. However, our results must be interpreted with caution given the well accepted limitations of use of historical control data and multiple non-randomized comparison groups. While the multivariate analysis can control for some of these differences and the main known MTCT risk factors, it was not possible to control for all possible confounders. Anyway, using the combined outcome of HIV-free survival, our study helps assessing the public health effectiveness of the combination of scARV regimens with alternatives to prolonged breastfeeding and answering to the following issues. Do alternatives to prolonged breastfeeding (formula feeding and shortened breastfeeding) decrease MTCT versus prolonged breastfeeding after using scARV regimens without increasing death? Does formula feeding prevent more infections than 4-month breastfeeding after using scARV regimens? What is the effectiveness on MTCT of adding 3TC to ZDV+sdNVP?

First, we demonstrated that both formula feeding from birth and shortened breastfeeding, combined with any scARV combined regimens decreased significantly the 18-month risk of HIV transmission when compared to long-term breastfeeding combined with ZDV (data not shown). Using the reference group of children exposed to maternal ZDV regimen and prolonged breastfeeding, our results confirm the greater effectiveness of ZDV+sdNVP and ZDV+3TC+sdNVP combined with any infant feeding option (formula-feeding or shortened breastfeeding) in increasing HIV-free survival, after adjustment on other MTCT determinants, with significant reductions ranging from 51% to 73%, except for the shortened breastfed ZDV+sdNVP cohort comparable to the reference group. To formally distinguish the effect of the postnatal interventions from the scARV intervention, a reference group of children exposed to ZDV and formula-feeding would have been necessary. However, they constituted a sample too small to provide reliable information (n = 8), and we preferred to exclude them from the analysis.

These results confirm our previously published 6 weeks transmission analysis [8]. The most effective of these scARV strategies could thus lead to residual long-term MTCT risks lower than 5%, in children born to mother not fulfilling eligibility criteria for ART and even after exposure to shortened breastfeeding. To our knowledge, this is the first time that such a PMTCT long-term outcome is reported in breastfeeding populations, and these encouraging results are close to those observed in developed countries with more complex PMTCT strategies. We can therefore conclude that both postnatal interventions, formula feeding from birth and shortened 4-months breastfeeding, were effective in increasing HIV-free survival in this context. HIV-infected women should choose the one adapted to their individual situation.

Second, within the Ditrame-Plus cohort, formula feeding may be slightly better than shortened 4-months breastfeeding combined with scARVs but the difference was not statistically significant.

Third, when considering the HIV-free survival outcome within the Ditrame-Plus cohort, the 3TC-based strategy was not significantly protective, just below the type-1 error level, although there was a tendency, probably by lack of statistical power. In fact, the treatment duration differed also substantially between these two cohorts because the scARV was initiated earlier among women treated with ZDV+3TC+sdNVP (32 weeks) than in women receiving ZDV+sdNVP (36 weeks). As a conclusion, we were not able to distinguish the treatment effect from the duration effect, but we conclude to an overall effectiveness of the 3TC strategy. The longer the duration of the pre-partum ZDV+3TC+sdNVP prophylaxis, the lower the 18-month MTCT risk: this dose-effect relationship argues for a plausible causal relationship between the cumulative drugs intake and their effect in reducing durably the maternal plasma HIV viral load, a strong determinant of mother-to-child transmission [29]. This effect was already reported in the Thai ZDV+sdNVP trial with a significant higher protective effect on HIV infection with longer ARV prophylaxis in a non breastfeeding population [30]. The 18-month residual MTCT rate in the Ditrame-Plus cohort exposed to ZDV+sdNVP and formula feeding was higher (9.4%) than the one measured in Thailand reaching 2% at 6–8 weeks [30]. This discrepancy is probably related to the pre-partum duration of ARV prophylaxis, beginning at 28 weeks of gestation in the Thailand trial, i.e., four to eight weeks earlier than in Ditrame-Plus, enough to lower efficiently the maternal HIV plasma viral load. As a result, the earlier the maternal scARV regimen begins, the lower the peripartum transmission rate is. This is in agreement with the current 2006 WHO PMTCT guidelines [31].

Thus, the short-course peripartum ZDV+3TC regimen combined with sdNVP from 32 weeks would be proposed as a valuable option, particularly in women who choose to breastfed, considering also its ability in minimizing the emergence of NVP-resistance [32]. However this combination was also associated with the risk of emergence of 3TC resistance, 15% in our cohort, with potentially adverse consequences for maternal health [32], [33]. Indeed, we reported that 3TC-resistance acquired after the exposition to a PMTCT regimen is responsible for a significant virological failure of the subsequent maternal response to HAART [33]. The longer the duration of 3TC-exposure, the higher the risk of emergence of resistance [34]. So, we conclude that ZDV+3TC regimen combined with sdNVP should be kept as a late indication after 36 weeks. Consequently, our findings could have implications on the 2006 WHO recommendations. If HIV-infected pregnant women are eligible for HAART, they should receive it since with very low MTCT rates can be achieved [35]. If they are not eligible for HAART or if it is not available, then the strategy could be as follows. If she is identified before 36 weeks of gestation, the ZDV+sdNVP strategy should be proposed with a tail of 3 days ZDV+3TC to reduce NVP-resistance. If she is identified from 36 weeks of gestation, the ZDV+3TC+sdNVP strategy should be proposed with a tail of 3 days ZDV+3TC to reduce NVP-resistance. Beyond any of these peri-partum interventions, post-partum interventions should be associated.

The long-term effectiveness of peri-partum and post-partum combined interventions needs to be balanced with their maternal social acceptability, and their impact on 2-year health outcomes in children: postnatal transmission, morbidity and mortality. The cost issue of the postnatal interventions also needs to be addressed. In the Ditrame-Plus cohort, we pragmatically used multiple judgment criteria to assess infant feeding options. First, there was a high acceptability of both alternatives to prolonged breastfeeding leading to a real opportunity of controlling for the risk of HIV transmission through breast-milk. Formula feeding was acceptable for half of the women with less than 15% of failures (defined as breastfeeding at least one) observed until one year, leading to few mixed-fed children [10]. Among breastfeeding mothers, the duration of breastfeeding was reduced to four months in median [9]. When assessing HIV-free survival outcomes in formula-fed children compared to the long-term breastfed ones, our observation strengthens the safety results we had reported previously, with two-year morbidity and mortality rates similar between shortened breastfed and formula-fed children [11]. These two groups of children did not have either any excess mortality compared to the long-term breastfed children of the Ditrame cohort [11]. In this “long-term” breastfed group, the breastfeeding duration was shorter than practices usually reported in Africa (the median duration was 7.3 months), but we estimated the difference in HIV-transmission or death in a very conservative way. In the Mashi trial, HIV-free survival outcomes were comparable between formula fed children and breastfed ZDV-exposed children: breastfeeding after ZDV prophylaxis was not as effective as formula feeding in preventing postnatal HIV transmission, but was associated with a lower mortality rate than formula feeding at 7 months [36]. In our study, we conclude to the superiority of the effectiveness of the combination of peri-partum interventions with alternatives to prolonged breastfeeding.

However, it is crucial to bear in mind the conditions required to consider safely alternatives to breastfeeding in an African context: access to clean water as it was the case in this urban setting, nutritional information and formulation of the maternal choice during the prenatal period, equipment and formula feeding given for free for at least for nine months, regular postnatal follow-up with nutritional counseling and repeated growth measurements. Given these conditions, it was appropriate to promote infant feeding options to women in our settings. The most appropriate infant feeding option for an HIV-infected mother should depends on her individual circumstances, including her health status, the local situation and her risk of stigmatization, but should take greater consideration of the health services available and the counseling and support she is likely to receive, as recently recommended by WHO [37]. Other postnatal interventions need to be further investigated to prevent MTCT in Africa, such as maternal ART while breastfeeding, either restricted to women needing it for their own health or prescribed to all women during breastfeeding with early cessation from six months [38]. Research studies are needed to fully understand the impact of maternal ARV treatment on the reduction of breastfeeding HIV-1 transmission, but also on infant toxicity and on acquisition of HIV-1 resistance mutations among infected infants [38], [39]. Alternatively, prolonging maternal and/or infant post-exposure prophylaxis with scARV regimens deserves proper evaluation.

Altogether, we demonstrate in this large African urban cohort that the risk reductions obtained with these relatively simple and affordable scARV drug regimens combined with acceptable alternatives to breastfeeding are getting closer to those observed in developed countries, with a sustained long-term effect even in shortened breastfed children. A decision analysis could be helpful in guiding recommendations on the safest and best infant feeding modalities according to the different African contexts combined with the full spectrum of ARV strategies, including ART for those in need.
